# Inflammatory Alterations of the Extracellular Matrix in the Tumor Microenvironment

**DOI:** 10.3390/cancers3033189

**Published:** 2011-08-09

**Authors:** Junko Iijima, Kenjiro Konno, Naoki Itano

**Affiliations:** 1 Department of Molecular Biosciences, Faculty of Life Sciences, Kyoto Sangyo University, Motoyama, Kamigamo, Kita-Ku, Kyoto 603-8555, Japan; E-Mail: iijima@cc.kyoto-su.ac.jp; 2 Department of Animal Medical Sciences, Faculty of Life Sciences, Kyoto Sangyo University, Motoyama, Kamigamo, Kita-Ku, Kyoto 603-8555, Japan; E-Mail: kkonno@cc.kyoto-su.ac.jp

**Keywords:** microenvironment, cancer progression, extracellular matrix, hyaluronan, inflammation

## Abstract

Complex interactions between cancer cells and host stromal cells result in the formation of the “tumor microenvironment”, where inflammatory alterations involve the infiltration of tumor-associated fibroblasts and inflammatory leukocytes that contribute to the acquisition of malignant characteristics, such as increased cancer cell proliferation, invasiveness, metastasis, angiogenesis, and avoidance of adaptive immunity. The microenvironment of a solid tumor is comprised not only of cellular compartments, but also of bioactive substances, including cytokines, growth factors, and extracellular matrix (ECM). ECM can act as a scaffold for cell migration, a reservoir for cytokines and growth factors, and a signal through receptor binding. During inflammation, ECM components and their degraded fragments act directly and indirectly as inflammatory stimuli in certain cases and regulate the functions of inflammatory and immune cells. One such ECM component, hyaluronan, has recently been implicated to modulate innate immune cell function through pattern recognition toll-like receptors and accelerate the recruitment and activation of tumor-associated macrophages in inflamed cancers. Here, we will summarize the molecular mechanism linking inflammation with ECM remodeling in the tumor microenvironment, with a particular emphasis on the role of hyaluronan in controlling the inflammatory response.

## 1. Introduction

Cancers develop in a complex and dynamic microenvironment. During cancer initiation and progression, cancer cells receive signals from the tumor microenvironment and communicate bilaterally with host stromal cells [1-4]. These cellular communications extensively alter the cellular and molecular composition of a particular tumor microenvironment. In breast cancer, dynamic changes in the microenvironment are characterized by formation of tumor stroma containing abundant fibrous components as well as by active recruitment of inflammatory cells during the progression from mammary gland hyperplasia to adenoma formation and eventually to cancer [5,6]. It is becoming increasingly clear that the inflammatory changes occurring in the tumor microenvironment are closely linked with the initiation, promotion, and progression of tumorigenesis, and thereby have become the focus of intense research [7,8]. Previous studies have highlighted the important roles of inflammatory cells, especially macrophages, in the development and progression of inflammation-associated cancers [9-11]. Infiltrating pro-inflammatory immune cells within cancers contribute to the maintenance of cytokine-enriched circumstances, which leads to further remodeling of the tumor microenvironment. The first part of this review summarizes our current understanding of the mechanism by which inflammatory reactions alter tumor microenvironments to facilitate malignant transformation and progression. Furthermore, as it has recently become evident that the ECM components and their proteolytic fragments have a considerable impact on the inflammatory response [12], the latter part of this review will focus on the linkage between inflammation and ECM remodeling in the tumor microenvironment.

## 2. Inflammatory and Innate Immune Responses in the Tumor Microenvironment, a Brief Overview

**Figure 1.**
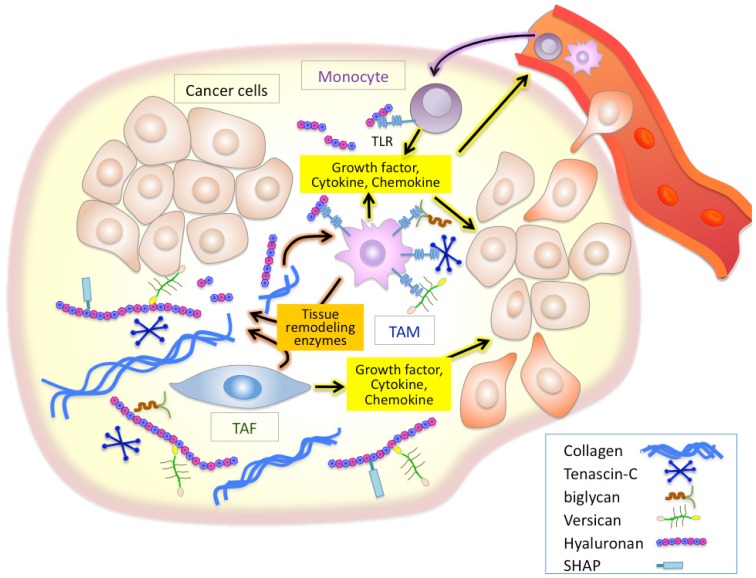
Schematic of the inflammatory tumor microenvironment. Inflammatory cells produce inflammatory mediators, including cytokines, chemokines, and tissue remodeling enzymes, which in turn amplify and perpetuate the inflammatory cascade. Extracellular matrix (ECM) components serve as a structural scaffold for inflammatory cell infiltration as well as a reservoir for cytokines and growth factors. Enzymatic degradation of ECM components generates bioactive fragments that play key roles in controlling inflammatory processes. Tenascin-C, biglycan, versican, and hyaluronan initiate toll-like receptor (TLR)-mediated innate immune responses.

Inflammation is a well established characteristic of cancer. Chronic inflammatory conditions are considered to increase cancer risk and accelerate the development and progression of inflammation-associated cancers. As cancers progress, stromal and inflammatory cells, both of which are recruited into cancer sites, stimulate inflammatory cascades in the tumor microenvironment. Tumor-recruited fibroblasts called tumor-associated fibroblasts (TAFs) are the main cellular components of the stroma of many solid tumors [13,14]. TAFs are known to supply many inflammatory mediators including a variety of cytokines, growth factors, tissue remodeling enzymes such as matrix metalloproteinases (MMPs), and ECM components [15-18], all of which modulate the tumor microenvironment to aid the active mobilization of the inflammatory cells [19-23] (Figure 1). Accumulating evidence indicates that macrophages are important determinants of the inflammatory response in most cancers [9-11]. Studies in *op*/*op* mice deficient for the macrophage growth factor CSF-1 have provided strong supporting findings on the close linkage between macrophages and tumor progression [24]. Macrophages are critical effecter cells in the innate immune response, and their lineage comprises a very diverse cell system with respect to phenotypic and functional properties; classical macrophages, termed M1, are capable of efficiently killing tumor cells, while M2 macrophages are immunosuppressive and pro-tumorigenic [9-11]. Tumor-associated macrophages (TAMs), which have little cytotoxicity for tumor cells and can actually promote tumor cell proliferation, resemble M2-polarized macrophages. Recent reports have established that the acquisition of pro-tumorigenic functions by TAMs is driven by various cytokines and signals expressed within the tumor microenvironment, thus powering further dynamic change of the tumor microenvironment [19,21]. It is therefore believed that TAFs and TAMs play crucial roles in inflammatory alterations of tumor microenvironment in a cooperative fashion (Figure 1).

## 3. ECM Remodeling in Tumor Microenvironment

The hallmarks of chronic inflammation include increased infiltration of activated inflammatory cells, mediator release, and turnover of ECM components. An aberrant turnover of ECM components is often detected in cancers, particularly under inflammatory conditions [25,26]. ECM components serve as a structural scaffold for inflammatory cell infiltration as well as a reservoir for cytokines and growth factors [27]. In addition, certain ECM molecules can act indirectly as inflammatory stimuli by inducing the expression of proinflammatory genes. The degradation of ECM components gives rise to the generation of bioactive fragments that play key roles in controlling numerous events, including tissue remodeling, inflammation, angiogenesis, tumor growth, and metastasis [28]. Matrikines, which are fragments obtained by proteolytic cleavage of ECM constituents, have biological functions distinct from those of parental proteins and act directly as inflammatory stimuli in certain cases [29,30]. Proteolytic fragments generated from collagen types I and IV, fibronectin, laminins, elastin, entactin/nidogen, and thrombospondin-1/2 have been shown to act as chemoattractants for inflammatory cells. In addition to the direct action of these degraded fragments, indirect effects on inflammatory responses are also seen via specific protease and cytokine gene induction that are necessary for matrix remodeling and inflammation.

There is growing evidence that ECM molecules can activate the innate immune system as endogenous antigens in a manner analogous to pathogen-associated molecular patterns [31-33]. Of such ECM molecules, tenascin-C, biglycan, versican, and hyaluronan have been demonstrated to initiate toll-like receptor (TLR)-mediated innate immune responses [34-38] (Figure 1). Tenascin-C is a proinflammatory extracellular glycoprotein whose expression is specifically and rapidly induced in response to tissue injury [39]. Tenascin-C-mediated chronic inflammation is clearly associated with the symptoms of rheumatoid arthritis [39]. One study also suggested that tenascin-C induces cytokine synthesis in macrophages and synovial fibroblasts as an endogenous activator of TLR4 in arthritic joint disease [34]. Since elevated tenascin-C expression is often observed in chronic inflammation of tumor stroma, an analogous mechanism may play an important role in inflammation in the tumor microenvironment [39]. Biglycan is a small leucine-rich proteoglycan with a core protein and one or more chondroitin/dermatan sulfate chains. Recently, biglycan has been reported to bind to the endocytic mannose receptor, a pattern recognition receptor expressed on macrophages and dendritic cells (DCs) [40]. Binding of biglycan significantly increases IL-10 production and decreases IL-12 in lipopolysaccharide-maturing DCs, suggesting a down-regulation of Th1-polarized immune responses. Schaefer *et al.* has demonstrated that biglycan activates intracellular signaling pathways through TLR2 and TLR4 in macrophages, eventually leading to an enhanced synthesis of proinflammatory TNF-α and MIP-2 [35].

**Figure 2.**
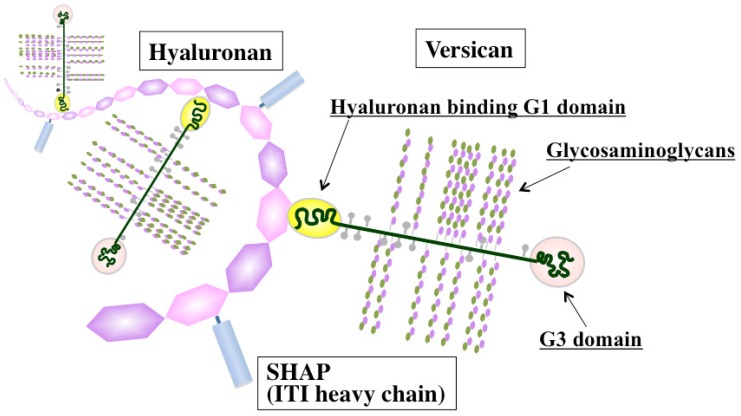
Schematic of the hyaluronan-enriched extracellular matrix. Hyaluronan assembles ECM through association with its binding partners, such as versican proteoglycan and SHAP, and serves as a structural scaffold for inflammatory cells.

Versican, a large chondroitin sulfate proteoglycan, is structurally composed of a hyaluronan-binding G1 domain at the *N*-terminus, glycosaminoglycan attachment regions, and a C-terminal G3 domain containing two epidermal growth factor (EGF)-like repeats, a C-type lectin-like motif, and a complement binding protein (CBP)-like motif [41] (Figure 2). Versican is involved in ECM assembly via the association of these specific domains with various other ECM constituents, such as hyaluronan, tenascin-C, fibronectin, fibulin-1, and fibrillin. Through interactions with ECM molecules, versican regulates many cellular processes of adhesion, migration, proliferation, apoptosis, and angiogenesis, all events implicated in tumor progression and metastasis. Elevated expression of versican both in tumor stroma and in cancer cells is frequently associated with poor outcome in breast, brain, ovary, and prostate cancers, sarcoma, and mesothelioma [42]. Due to its interactions with CXC and CC chemokines, versican may also act as a chemokine reservoir and facilitate the recruitment of inflammatory cells via chemokine release [43].

Another suspected role of versican lies in its ability to mediate the retention of inflammatory leukocytes through interactions with cell surface CD44 and L-selectin receptors [44]. Weight and colleagues have reported an important role for versican in the hyaluronan-dependent binding of monocytes to the ECM of lung fibroblasts stimulated with poly I:C, although the responsible receptor has not been identified yet [45]. By revealing the molecular cues that promote cancer metastasis, Karin *et al.* recently uncovered a new inflammatory pathway in which versican activates macrophages via TLR2 and its co-receptors TLR6 and CD14 [36]. In that study, they further showed that ligation of TLR2 by versican elicits the production of proinflammatory cytokines, providing a positive link between inflammation and cancer metastasis.

**Figure 3.**
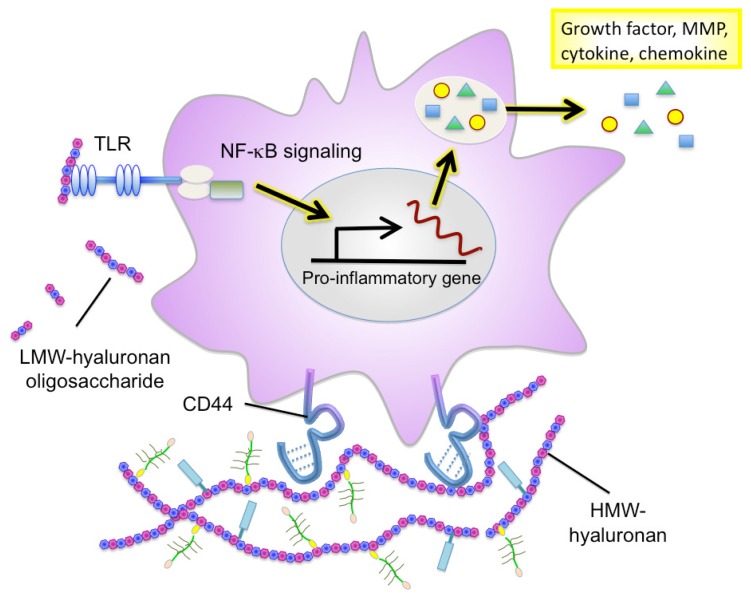
Inflammatory responses of monocytes/macrophages to hyaluronan. During tumor progression, high molecular-weight (HMW) hyaluronan forms part of the tumor microenvironment by linking its binding partners into macromolecular aggregates. The hyaluronan-enriched tumor microenvironment accelerates recruitment and activation of monocytes/macrophages. Low molecular-weight (LMW) hyaluronan generated by hyaluronan fragmentation accelerates inflammatory responses via TLRs on macrophages and regulates inflammatory gene expression.

Hyaluronan serves as an attachment ligand for receptors on inflammatory cells and is partly responsible for leukocyte retention in the inflammatory response [46]. CD44 is one such receptor and has been shown to participate in monocyte/macrophage retention and activation in inflammatory sites [47] (Figure 3). Similarly to other ECM molecules, hyaluronan degradation products have the ability to induce specific gene expression programs for proteases and cytokines that are necessary for inflammation and matrix remodeling. Several recent studies have shown that hyaluronan fragments activate innate immune responses by interacting with TLR2 and TLR4 and inducing inflammatory gene expression in a variety of immune cells [37,38,48] (Figure 3). In light of this, we will focus the latter half of this review on the key role of hyaluronan in facilitating the inflammatory circuit in the tumor microenvironment.

## 4. Hyaluronan Accumulation in Cancers

Hyaluronan is the main component of the tumor microenvironment and has thus become an increasingly important target for cancer therapy [49]. While hyaluronan is essential for the construction of normal tissue architecture, it is also known as a polysaccharide closely related to cancer [50]. The association between a rise in hyaluronan and cancer dates back to the 1950s, when a study first reported increased hyaluronan production in a mesothelioma [51,52]. Since then, hyaluronan has been accepted as a significant prognostic factor in many types of cancers, and high levels of this molecule are correlated with a poor prognosis [53,54]; in several studies, the proportion of hyaluronan-positive cells and intensity of hyaluronan staining in breast cancer tissues were closely correlated with a poor survival rate and prognosis [55]. In colorectal cancers, the 5-year survival rate of patients with a large fraction of hyaluronan-positive cancer cells was reported to be only 20% [56]. Poorly differentiated or high-grade tumors generally have more stromal hyaluronan than well-differentiated tumors, and multivariate analyses have confirmed the rate of positive staining for hyaluronan in tumor stroma to be an independent poor prognostic factor. In ovarian cancers, the hyaluronan level in tumor stroma was correlated with the degree of cancer cell undifferentiation and cancer stage [57]. Therefore, hyaluronan, and especially stromal hyaluronan, may be an important factor determining the malignant characteristics of cancers.

## 5. Biosynthesis, Catabolism and Physiopathological Functions of Hyaluronan

**Figure 4.**
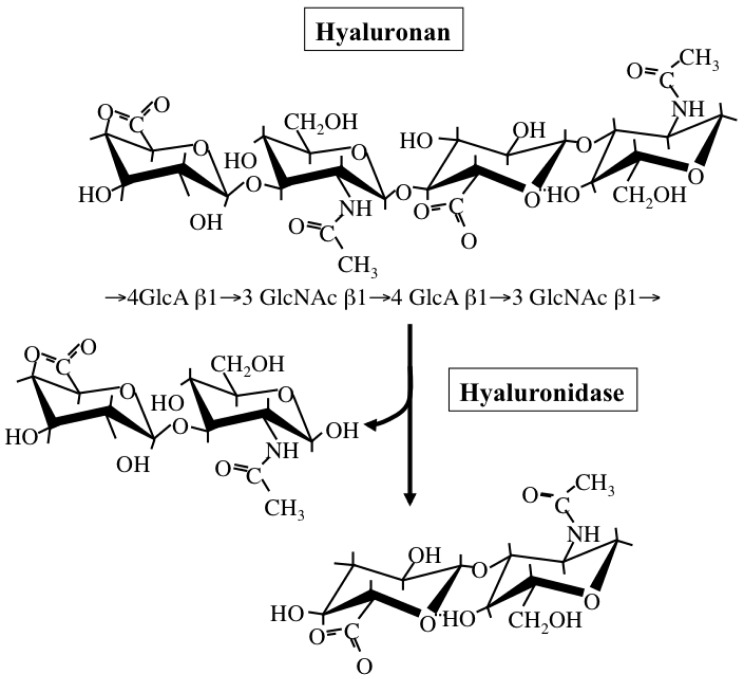
Hyaluronan structure and degradation. Hyaluronan is composed of repeating disaccharide units of *N*-acetylglucosamine (GlcNAc) β(1→4)-glucuronic acid (GlcA) β(1→3). Hyaluronidase cleaves *N*-acetylglucosamine β(1→4) glycoside bonds in hyaluronan.

Hyaluronan is a simple polysaccharide molecule composed of repeating disaccharide units of alternating *N*-acetylglucosamine and glucuronic acid (Figure 4). The dynamic turnover of hyaluronan is tightly balanced by its synthesis and degradation to maintain a specific concentration and chain length in tissues [58]. Hyaluronan synthesis is catalyzed by one of three isoforms of the HAS enzyme: HAS1, HAS2, and HAS3. Structurally, all HAS enzymes are integral membrane proteins composed of multiple membrane-spanning regions with hydrophobic amino acid clusters and a large cytoplasmic loop. In this loop, there are two catalytic sites participating in the transfer of UDP-GlcNAc and UDP-GlcA substrates. These isoforms however differ from one another in the length of sugar chains synthesized and ECM-forming ability [59]. Regarding the subcellular localization of HAS enzymes, a recent study has also proposed that latent enzymes reside around the endoplasmic reticulum (ER) and nuclear membrane and synthesize cable-like hyaluronan structures upon particular cellular stresses, such as ER stress [60]. This provides evidence for multiple regulatory mechanisms of HA biosynthesis, where structurally different HA molecules are synthesized in different subcellular compartments depending on cellular state.

Hyaluronan catabolism is predominantly regulated by several hyaluronidases (Figure 4), which are classified as endo-β-*N*-acetylglucosaminidases according to their hydrolytic mechanisms [61]. Six structurally homologous hyaluronidase-related genes (HYAL-1, -2, -3, -4, -P1, and PH-20) have been identified so far in mammals [61]. Among them, the main tissue hyaluronidases HYAL-1 and -2 regulate hyaluronan degradation in a sequential series of catabolic reactions. HYAL-2 degrades high molecular-weight hyaluronan into fragments of up to 20 kDa, HYAL-1 breaks down hyaluronan into fragments of up to 800 Da. Since gene expression of HYAL-1 and -2 is up-regulated under pathological conditions, hyaluronidase-mediated hyaluronan degradation appears to occur in subjects with cancer and chronic inflammation.

The physiopathological functions of hyaluronan vary in a manner dependent on its sugar chain length [62]. High molecular-weight (HMW) hyaluronan assembles the ECM in association with binding molecules (Figure 3). This type of hyaluronan ECM is believed to maintain tissue structure and serve as a scaffold for cell adhesion and migration. HMW hyaluronan exhibits space-filling and water-retention properties and functions to modulate the pericellular ECM microenvironment. Alternatively, oligosaccharides produced from the catabolic activity of hyaluronidase diffuse through tissues and bind to hyaluronan receptors on adjacent cells, thereby acting as intracellular signals such as NF-kB and Erk [63]. The promotion of tumor angiogenesis and induction of inflammation by hyaluronan oligosaccharides are well defined, as shown in Table 1.

## 6. Aberrant Hyaluronan Turnover and Function in Cancers

The rates of hyaluronan synthesis and degradation are much higher in cancers than in normal tissues [50]. The malignant transformation of cells frequently impairs regulation of hyaluronan synthesis and leads to excessive hyaluronan production [53,78]. Studies using several cancer cell lines reported that HAS expression was increased in cancer cells whose ability to produce hyaluronan was enhanced [79,80]. Furthermore, amplification of the genomic region that includes the HAS2 gene has also been found with high frequency in prostate cancer [81]. Although not directly proven, HAS gene amplification may be directly linked to increased hyaluronan synthesis in cancer cells. These results suggest that abnormal hyaluronan accumulation in cancer tissue is the result of transformed cells acquiring a high hyaluronan-producing ability associated with increased HAS expression. In addition to excess hyaluronan production in cancer cells, TAFs and other stromal cells are also considered to contribute significantly to the accumulation of hyaluronan [82]. In cancer tissues, gene expression of hyaluronan synthases in cancer cells and fibroblasts is elevated following exposure to various growth factors and cytokines, resulting in a marked increase in hyaluronan synthesis [82-84]. Pathological analysis of clinical samples has demonstrated that the increased expression of HAS1 among the three HAS isoforms in human colorectal cancer tissues is correlated with lymph node metastasis [85]. Although a close correlation between HAS gene expression and tumor formation and metastasis has been suggested by many studies using cultured tumor cells [86,87], exceptions have also been observed. Enegd *et al.* reported that the forced expression of HAS2 in glioma cells resulted in the suppression of tumor formation, and postulated that a balance between hyaluronan degradation by hyaluronidase and synthesis by HAS was responsible for tumor formation [88]. These observations therefore suggest that hyaluronan promotes tumor progression at a specific concentration range and sugar-chain length, but inhibits tumor formation at other concentrations or sugar-chain lengths.

The expression of such hyaluronidases is up-regulated in certain cancers [89-91]. Pathological analysis of clinical samples has shown that HYAL-1 expression is a good prognostic indicator of prostate and bladder cancers [89], while the analysis of breast cancer cell lines has revealed that highly invasive cancer cells express high levels of HYAL-2 [92]. The association between hyaluronidase and tumor formation has been directly confirmed by the fact that HYAL-2 overexpression in mouse astrocytoma promotes the formation of tumors [93]. On the other hand, similarly to HAS, hyaluronidase exhibited a completely opposite effect in some cases; a human breast cancer transplanted into immunodeficient (SCID) mice markedly regressed after intravenous administration of testicular hyaluronidase [94], and HYAL-1 overexpression in a colorectal cancer model inhibited tumor formation [95]. These conflicting results support the notion of a delicate balance between hyaluronan degradation and synthesis in tumor development.

In advanced cancer, therefore, aberrant synthesis and degradation of hyaluronan result in the formation of an extremely unusual microenvironment characterized by the accumulation of HMW hyaluronan and excessive hyaluronan oligosaccharides, which may facilitate the malignant transformation and survival of cancer cells.

## 7. The Roles of Hyaluronan in Inflammation

Recent studies have firmly implicated hyaluronan in the regulation of immune and inflammatory cell responses. Hyaluronan synthesis is regulated by numerous pro-inflammatory factors and cytokines. Hyaluronan oligosaccharides act on monocytes/macrophages to differentiate them into M2 macrophages [96], suggesting that they can regulate polarization of myeloid lineages by affecting the balance of Th1/Th2 cytokines in local environments. Emerging evidence further points to an important role of hyaluronan fragments in the induction of many inflammatory cytokines (IL-8, IL-12, and TNF-α) and chemokines (MIP-1α, MIP-1β, KC, RANTES, MCP-1, and IFN-inducible protein-10) in a variety of immune cells [66,72,73] (Figure 3). Furthermore, hyaluronan fragments serve as an endogenous ligand for TLRs, which is crucial for the activation of innate immune cells [37,38,48]. Interestingly, TLR-mediated activation is hyaluronan size-dependent and is only seen in the presence of smaller molecule fragments 4–16 oligosaccharides in size.

Considerable information has been accumulated on the ability of hyaluronan-associated molecules to modulate hyaluronan functions affecting immune and inflammatory responses (Figure 2). de la Motte *et al.* showed that hyaluronan and its associated molecules formed cable-like ECM structures that were involved in the adhesion and recruitment of monocytes through association with hyaluronan receptor CD44 [97]. Several hyaluronan-binding partners, such as SHAP, tumor necrosis factor stimulated gene 6 (TSG-6), and versican, are located along the strands of cable-like ECM containing hyaluronan and are also expected to play crucial roles in structure formation [98]. SHAP, a serum-derived hyaluronan-associated protein, participates in the construction of such cable-like structures and enhances hyaluronan-mediated leukocyte adhesion after forming a complex with hyaluronan [99] (Figure 2). SHAP corresponds to the heavy chains of the plasma inter-α-trypsin inhibitor (ITI) family and covalently binds to hyaluronan via a unique ester bond. The transfer of SHAP to hyaluronan is mediated by TSG-6 through a hyaluronan-binding link module [100]. Serum levels of SHAP-hyaluronan complexes have been reported to be closely associated with the patho-physiology of inflammatory diseases, such as rheumatoid arthritis, and correlated with the clinical stages of chronic hepatitis [101,102]. A similar observation was also made in ovarian cancers, where SHAP serum levels were correlated with clinical outcome [103]. Since hyaluronan and versican can stimulate macrophage cytokine and chemokine production in a TLR-dependent manner, the co-localization of these macromolecules may imply their cooperative action in the innate immune response. Our recent study demonstrated the preferential engagement of immunosuppressive M2 macrophages in a hyaluronan- and versican-rich stromal microenvironment [104]. The effects were typically observed only with the hyaluronan-versican complex, supporting their cooperative action as modulators of innate immunity. Taken together, these observations indicate that the hyaluronan-enriched tumor microenvironment regulates innate immune and inflammatory responses by enhancing the recruitment of innate immune cells and modulating inflammatory gene expression.

## 8. Conclusions and Future Prospects

Recent findings have shed new light on the inflammatory alterations that take place in the tumor microenvironment. Emerging evidence supports the view that ECM is not only remodeled as a result of inflammation, but also directly and indirectly influences the inflammation circuit. During inflammation, ECM components and their fragments play substantial roles in inflammation cascades; matrikines derived proteolytically from ECM components act directly as inflammatory stimuli. Tenascin-C, proteoglycans, and hyaluronan initiate TLR-mediated inflammation reactions. These components also have important functions as reservoirs for inflammatory cytokines, inducers of gene expression, and modulators of biological activity. It therefore appears that the enrichment of these ECM molecules in the tumor microenvironment is a sign of inflammation. Since the inflammatory matrix provides an environment amenable to the acquisition of malignant characteristics for tumor cells, further elucidation of the precise mechanisms controlling inflammatory matrix reconstruction will allow us to develop novel and promising therapeutic strategies to prevent the aberrant matrix turnover that is associated with cancer cell growth, invasion, metastasis, and angiogenesis.

## Figures and Tables

**Table 1.** Inflammatory actions of hyaluronan and its oligosaccharides.

**Hyaluronan length (mers or kDa)**	**Inflammatory-related functions of hyaluronan**	**References**
4–6 mers	Activation of NF-ĸB and upregulation of MMP expression	[64,65]
4–16 mers	Activation and maturation of DCs Induction of cytokines and chemokines	[37,66-68]
6–32 mers	Stimulation of angiogenesis	[69-71]
40–800 kDa	Induction of proinflammatory cytokines and chemokines in macrophage Induction of iNOS and COX-2 in macrophage Activation of innate immunity	[48,72-77]

MMP: matrix metalloprotease; DC: dendritic cell; EC: endothelial cell.
